# Primary Gastric Lymphoma: Clinicopathological Profile

**DOI:** 10.5005/jp-journals-10018-1250

**Published:** 2018-05-01

**Authors:** Renuka Malipatel, Mallikarjun Patil, Patil Pritilata Rout, Marjorie Correa, Harshad Devarbhavi

**Affiliations:** 1 Department of Pathology, St. John’s Medical College, Bengaluru, Karnataka, India; 2 Department of Gastroenterology, St. John’s Medical College, Bengaluru, Karnataka, India

**Keywords:** Diffuse large B-cell lymphoma, Dyspepsia, Non-Hodgkin’s lymphoma, Primary gastric lymphoma.

## Abstract

**Introduction:**

Gastrointestinal tract (GIT) is the most common site of involvement of extranodal non-Hodgkin’s lymphoma (NHL). There is regional variation in anatomical distribution of extranodal NHL, stomach being the most common site followed by small intestine. Primary gastric lymphoma (PGL) predominantly involves the antrum and corpus of the stomach. It arises from mucosa-associated lymphoid tissue (MALT) and is of B-cell lineage and often associated with *Helicobacter pylori* infection. Primary gastric lymphoma often presents with nonspecific symptoms. The present study was undertaken to ascertain the clinicopathological characteristics of PGL at a tertiary care center in South India.

**Materials and methods:**

It is a retrospective study from 2006 to 2016. Patient’s data were obtained from institutional medical records. The histopathology slides were reviewed. The relevant immunohistochemistry (IHC) markers done were leukocyte common antigen (LCA), CD3, CD20, CD79a, CD10, Bcl-2, Bcl-6, CD5, Cyclin D1, CD138, and Ki-67. Correlating with the immunoprofile, further subtyping was done.

**Results:**

A total of 405 patients of NHL were seen during the study period, out of which 43 patients were PGL. There were 32 males and 11 females, with M:F of 2.9:1. The mean age at diagnosis was 58 years. Abdominal pain and new-onset dyspepsia were the commonly observed presenting symptoms. The common site of involvement was antrum (20). Diffuse large B-cell lymphoma (DLBCL) was the most common histological subtype. *Helicobacter pylori* infection was seen in 18 (41%) patients. Majority of the patients were in stages II and III.

**Conclusion:**

In our study, the initial presentation of PGL was with nonspecific symptoms like abdominal pain and new-onset dyspepsia. High degree of suspicion of such symptoms and biopsy of all suspicious lesions is essential for early detection. Diffuse large B-cell lymphoma was the most common histological subtype seen in our study.

**How to cite this article:** Malipatel R, Patil M, Rout P, Correa M, Devarbhavi H. Primary Gastric Lymphoma: Clinicopathological Profile. Euroasian J Hepato-Gastroenterol 2018;8(1):6-10.

## INTRODUCTION

Gastrointestinal tract is the most common site of involvement of extranodal NHL, stomach being the most common site of involvement , which accounts for less than 5% of primary gastric neoplasms.^1-4^ The involvement of stomach by lymphomas can be primary or secondary as a part of systemic lymphomas. The primary GIT lymphomas are defined as those in which involvement of the alimentary tract predominates or those with symptoms of GIT involvement on presentation.^5,6^ The diagnosis of PGL is often delayed due to nonspecific initial symptoms like vague abdominal pain and new-onset dyspepsia.^7^ Primary gastric lymphoma predominantly involves the antrum and corpus of the stomach, arise from MALT, and are of B-cell lineage.^8,9^ The different subtypes are DLBCL, extranodal marginal lymphomas of MALTs, Mantle cell lymphoma, follicular lymphoma, and very rarely T-cell lymphomas.^10-12^ The present study was undertaken to ascertain the clinicopathological characteristics of PGL at a tertiary care center.

## MATERIALS AND METHODS

The present study was retrospectively carried out in the Departments of Pathology and Gastroenterology, St. John’s Medical College, Bengaluru, Karnataka, India, from 2006 to 2016 (10 years). Patients’ data were collected from the institutional medical records. Patients presenting with GI symptoms or predominant tumor(s) in the GI tract were included as PGL based on Lewin et al definition.^5^ All patients underwent upper GI endoscopy (esophagogastroduodenoscopy) and gastric biopsy except one where laparotomy was done. The biopsies were processed in 10% formalin. Hematoxylin and eosin staining (H&E) was done on paraffin-embedded sections. The presence of *H. pylori* in the adjacent mucosa was noted. Immunohistochemistry was carried out manually on poly L-lysine-coated slides and controls run were satisfactory. Heat-induced antigen retrieval was done by pressure cooking the sections kept in Tris-ethylenediaminetetraacetic acid buffer. Avidin-Biotin peroxidase technique was used. The relevant IHC markers done were LCA, CD3, CD20, CD79a, CD10, Bcl-2, Bcl-6, CD5, Cyclin D1, CD138, and Ki-67. Correlating with the immunoprofile, further subtyping was done.

In all patients, physical examination, indirect laryngoscopy, complete blood count, liver function tests, lactate dehydrogenase, bone marrow examination, Chest X-ray, and computed tomography scan of the chest, abdomen, and pelvis were done for staging. Patients with systemic lymphomas involving the stomach were excluded from the study.

**Table 1:** **Table 1:** Various symptoms at presentation

*Symptom*		*No. of cases (n = 43)*	
Abdominal pain		15 (34.8%)	
New-onset dyspepsia		12 (27.9%)	
Gastric outlet obstruction		7 (16.27%)	
GI bleed		6 (13.95%)	
Dysphagia		2 (4.6%)	
Obstructive jaundice		1 (2.3%)	

**Table 2:** **Table 2:** Primary gastric lymphoma staging at presentation

*Stage*		*No. of cases (n = 43)*	
IE		1 (2.3%)	
I IE		26 (60.5%)	
III		9 (20.9%)	
IV		7 (16.27%)	

**Figs 1A to D:**
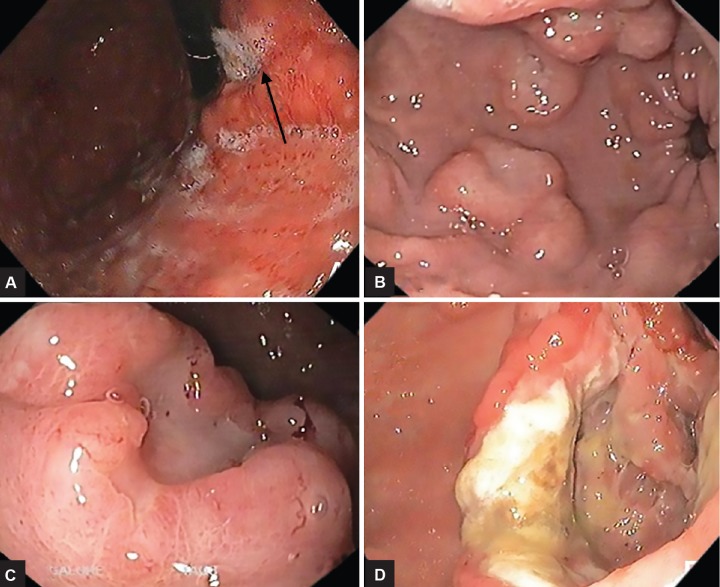
Endoscopic images showing thickened gastric folds (A), nodular lesions (B), and ulcerated lesions; (C) ulcerated lesion in the fundus of stomach; and (D) ulcerated lesion in the antrum

## RESULTS

During the study period between 2006 and 2016, total 405 patients of NHL were seen, out of which 43 patients were PGL, constituting 10.6% of all NHL. There were 32 males and 11 females, with M:F of 2.9:1. The mean age at diagnosis was 58 years.

**Figs 2A to F:**
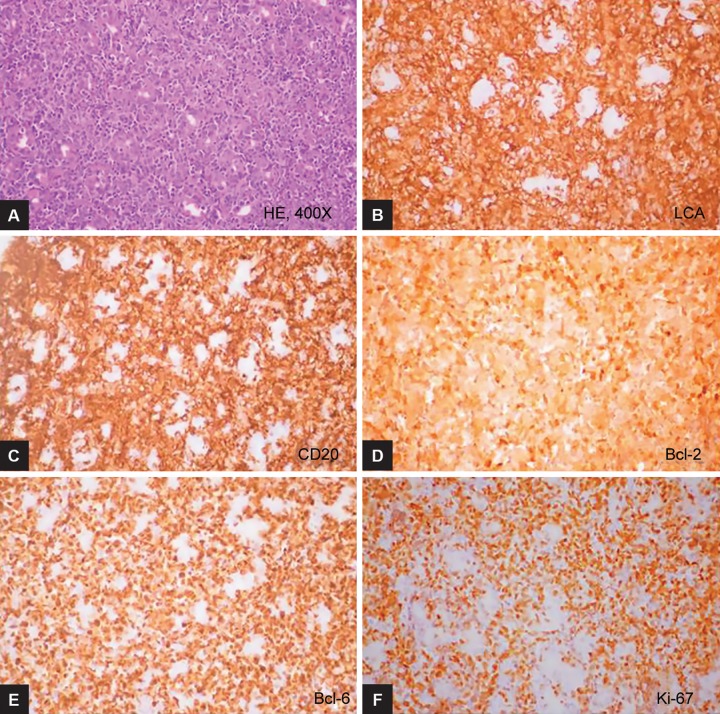
A case of DLBCL stomach showing sheets of monomorphic lymphoid cells (H&E stain, 400*) which express LCA, CD20, Bcl-2, Bcl-6. The i-67 proliferative index is 90%

**Figs 3A to D:**
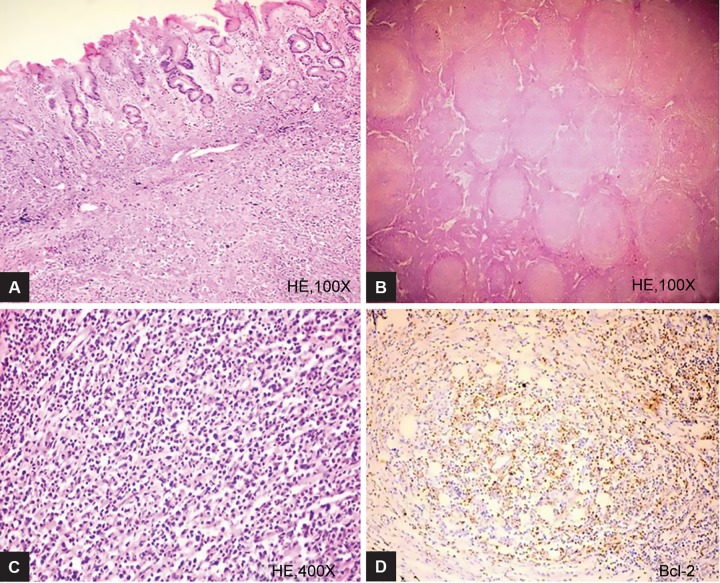
Resection specimen of stomach showing closely packed follicles of varying sizes (100*). These follicles are composed of centrocytes and centroblasts (higher magnification, 400*)

**Fig. 4:**
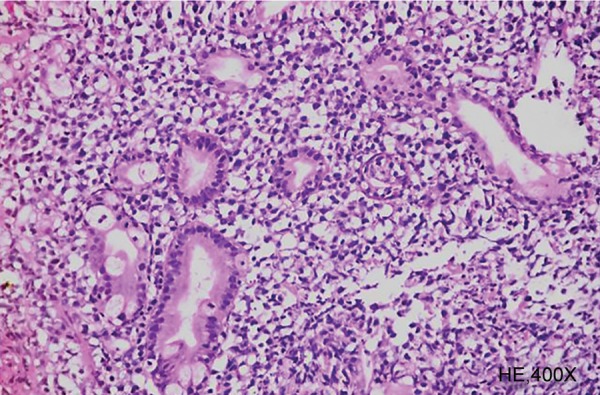
Stomach biopsy showing monomorphic lymphoid cell infiltrate and lymphoepithelial lesions

Abdominal pain and new-onset dyspepsia were the common presenting symptoms as shown in Table 1. The staging at the time of diagnosis was as shown in Table 2. The various sites of involvement were antrum (20), body (12), diffuse lesions (9), and fundus (2). The endoscopic appearances of PGL were as shown in Figure 1, ulcerated lesions (10), polypoidal lesions (19), thickened gastric folds (12), and erosions (2). The frequencies of various histological subtypes of PGL are listed in Table 3 and Figures 2 to 4. *Helicobacter pylori* infection was seen in 18 (41%) patients.

**Table 3:** **Table 3:** Primary gastric lymphoma histological subtypes

*Diagnosis*		*No. of cases (n = 43)*	
Diffuse large B-cell lymphoma		27 (62.8%)	
Low-grade marginal zone lymphoma of		15 (34.8%)	
MALT type			
Follicular lymphoma		1 (2.3%)	

## DISCUSSION

Gastrointestinal tract is the most common site of involvement for extranodal lymphoma, with stomach being the commonest. In our study, PGL constituted 10.6% of all NHL. The normal gastric mucosa is devoid of lymphatic tissue.^1^ The lymphoid tissue appears in the stomach in response to chronic inflammation induced by *H. pylori* infection. Most of the gastric lymphomas are thought to arise from the B-cell lineage of MALT. The presence of immunological evidence of *H. pylori* infection among patients with gastric lymphoma than matched controls^2,3^ and response to *H. pylori* eradication therapy^4,5^ proves the role of *H. pylori* infection in the pathogenesis of gastric lymphoma. In our study, *H. pylori* infection was seen in 41% of patients as compared with other studies which reported 75% by Shukla et al,^6^ 44% by Arora et al^7^ and 85% by Delchier et al.^8^ Low prevalence of *H. pylori* in our study could be due to the diagnosis of *H. pylori* infection made only on histology and serological test was not used.

Diffuse large B-cell lymphoma was the commonest histologic type seen in our study, which was similar to other studies from India and other parts of the world.^7,9,10^ Follicular lymphoma of the stomach is rare. In our study, there was one case of follicular lymphoma that presented with obstructive symptoms and required laparotomy.

As observed in other studies, PGL in our study was seen in elderly people, predominantly in males.^7,11,12^ Like in other studies the common presenting complaints were abdominal pain, new-onset dyspepsia, features of gastric outlet obstruction, anemia due to GI blood loss, jaundice, and dysphagia.^13-15^ None of the patients had B constitutional symptoms due to lymphoma. At the time of diagnosis majority of the patients were in stages II and III. Such late detection could be due to initial nonspecific symptoms.

## CONCLUSION

Primary gastric lymphomas present with initial nonspecific symptoms leading to delay in diagnosis. Hence, it is important to detect them at the earliest. Further studies on genetic and molecular profiling are necessary for understanding the pathogenetic mechanisms involved and for therapeutic and prognostic implications in different subtypes of PGL.
